# Solitary pulmonary metastasis from prostate sarcomatoid cancer

**DOI:** 10.1186/1477-7819-8-101

**Published:** 2010-11-19

**Authors:** Taichiro Goto, Arafumi Maeshima, Yoshitaka Oyamada, Ryoichi Kato

**Affiliations:** 1 Department of General Thoracic Surgery, National Hospital Organization Tokyo Medical Center, Tokyo, Japan; 2 Department of Pathology, National Hospital Organization Tokyo Medical Center, Tokyo, Japan; 3 Department of Respiratory Medicine, National Hospital Organization Tokyo Medical Center, Tokyo, Japan

## Abstract

**Background:**

Pulmonary metastasis from prostate cancer is considered to be a late event, and patients can be treated with chemotherapy or hormonal manipulation. However, there has been only a few reports on surgical resection for pulmonary metastasis from prostate cancer.

**Case Presentation:**

We present a surgical case of solitary pulmonary metastasis from prostate cancer. A 73-year-old man underwent pelvic evisceration for prostate cancer. Histopathological examination revealed a poorly differentiated adenocarcinoma with a sarcomatoid carcinoma component. During postoperative follow-up, chest computed tomography showed a nodular shadow in the lung, and thoracoscopic wedge resection of the lung was performed. Histopathological examination revealed a histological appearance similar to that of the prostate sarcomatoid carcinoma. This is the first reported case of solitary pulmonary metastasis from prostate sarcomatoid cancer.

**Conclusion:**

Isolated pulmonary metastasis from prostate sarcomatoid cancer is extremely rare, but surgery could be the treatment of choice.

## Background

Although prostate cancer often metastasizes to the lung, it usually metastasizes earlier to the bone [1]. Pulmonary metastasis from prostate cancer often shows a diffuse interstitial or multinodular pattern [2]. We report a rare surgical case of solitary pulmonary metastasis from prostate cancer.

## Case Presentation

**Figure 1:**
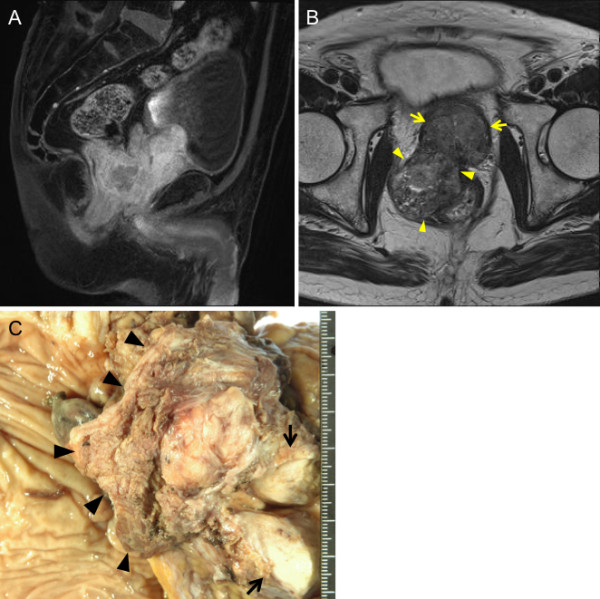
**Radiological and macroscopic findings of prostate cancer**. A-B, Pelvic MRI showed an enlarged prostate invading the bladder (arrow) and a polypoid mass invading the rectum (arrowhead)(A, T1-weighted sagittal view; B, T2-weighted axial view). C, Gross examination of the resected specimen revealed an enlarged prostate invading the bladder (arrow) and polypoid mass invading the rectum (arrowhead).

**Figure 2:**
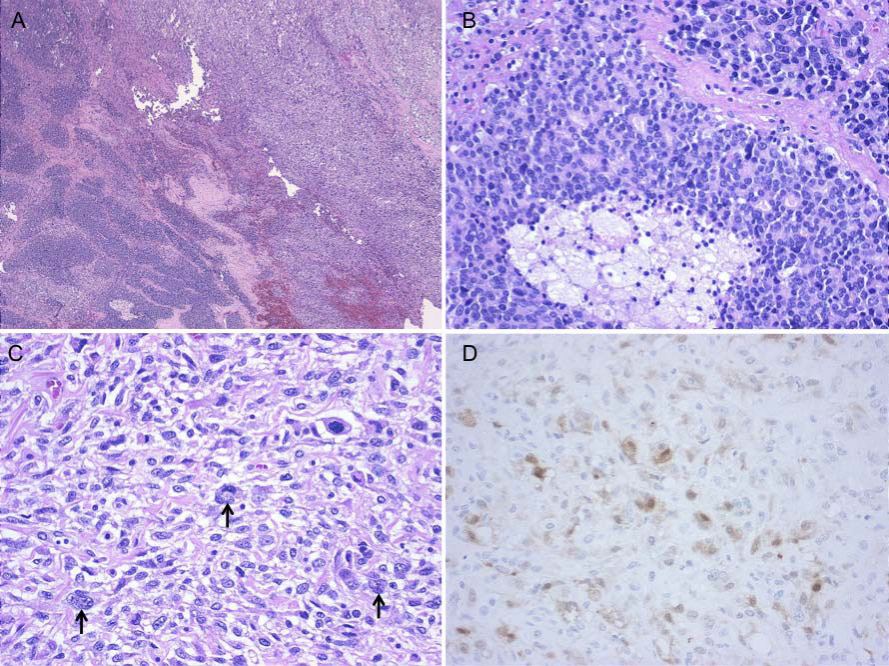
**Histological findings of prostate cancer**. A, The tumor was composed of a poorly differentiated Gleason 4/5 adenocarcinoma (lower left of photograph) and an undifferentiated sarcomatoid carcinoma (upper right of photograph). B, The intraprostatic tumor showing a mixture of cribriform structures and necrosis. C, The tumor in the area of posterior invasion. Arrows indicate multinucleated giant cells. D, Cytokeratin AE1/3 immunostaining of the sarcomatoid carcinoma component.

**Figure 3:**
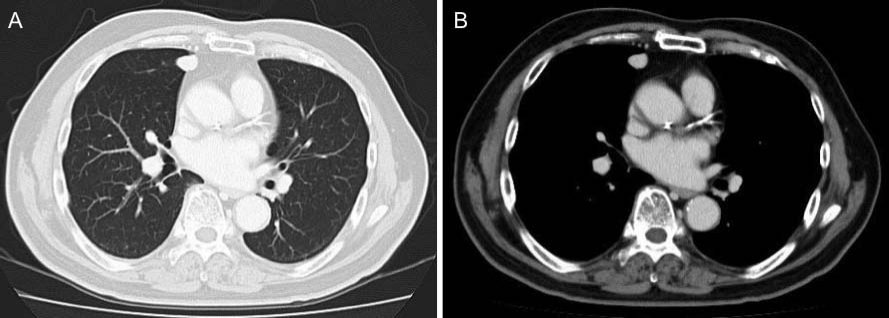
**CT findings of solitary pulmonary metastasis**. A-B, After pelvic evisceration, chest CT revealed a small nodular shadow (A, Lung window image; B, Mediastinal window image).

A 73-year-old man presented to the Department of Urology in our hospital with a chief complaint of nocturia. His past medical history was unremarkable. Pelvic computed tomography (CT) showed a large prostatic mass invading the rectum and bladder. The serum prostate-specific antigen (PSA) level was elevated to 14.37 ng/ml (normal range, 0.0-4.0 ng/ml). Prostate biopsy led to a diagnosis of poorly differentiated adenocarcinoma. In November 2008, the patient was started on maximum androgen blockade. The serum PSA level markedly declined, and has remained within the normal range since December 2008. Pelvic magnetic resonance imaging showed that the prostate decreased in size, but the mass invading the rectum tended to enlarge (Figure 1A, B). In April 2009, he underwent pelvic evisceration for the prostate cancer (Figure 1C). Histopathological examination revealed a poorly differentiated Gleason 4/5 adenocarcinoma in the prostate and an undifferentiated sarcomatoid carcinoma consisting of fascicularly arranged spindle cells in the area of posterior invasion (Figure 2A-C). This undifferentiated carcinoma component contained many multinucleated giant cells. Immunohistochemically, the undifferentiated carcinoma was positive for cytokeratin AE1/3, and negative for cytokeratin 7, cytokeratin 20, vimentin, and PSA (Figure 2D). During outpatient follow-up, the PSA level remained within the normal range, but 3 months after the pelvic evisceration, chest CT revealed a small nodular shadow in S3b of the right lung (Figure 3A). At the mediastinal window setting, the lung tumor showed a density similar to that of large vessels (Figure 3B). Despite the resumption of maximum androgen blockade, the tumor tended to enlarge. Positron emission tomography revealed an abnormal fluorodeoxy glucose uptake (SUVmax = 25.0) in the small nodular shadow, and no abnormalities elsewhere in the body. He was referred to our department for further evaluation and treatment. He was otherwise in good health. Blood chemistry data were unremarkable, and the tumor markers SCC, CEA, SLX, NSE, and PSA were all within normal limits.

**Figure 4:**
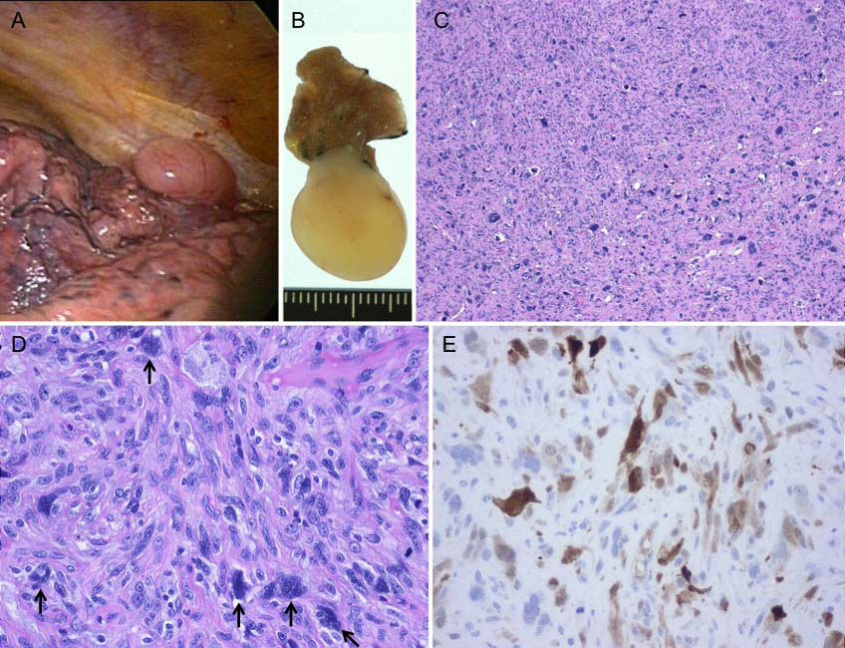
**Pulmonary metastasis of prostate cancer**. A, Gross appearance during thoracoscopic surgery. B, Gross appearance of the resected specimen. C-D, Histology of the lung tumor. Arrows indicate multinucleated giant cells. E, Cytokeratin AE1/3 immunostaining of the lung tumor.

Bronchoscopy was nondiagnostic, and thoracoscopic wedge resection of the lung was performed in December 2009 (Figure 4A). Pleural disease was not observed during thoracoscopy. The lung tumor, with a surface covered with the pleura, protruded into the pleural cavity in a polypoid fashion (Figure 4A, B). Histopathological examination revealed a histological appearance similar to that of the pre-existing undifferentiated sarcomatoid carcinoma, leading to a diagnosis of pulmonary metastasis from the prostate cancer (Figure 4C, D). Many multinucleated giant cells were also observed. As revealed by strong contrast enhancement on a preoperative CT scan, the tumor was histologically rich in microvessels. Immunohistochemically, it was positive for cytokeratin AE1/3, and negative for cytokeratin 7, cytokeratin 20, vimentin, and PSA (Figure 4E).

His postoperative course was uneventful, and postoperative adjuvant chemotherapy with cisplatin, ifosfamide, and adriamycin was administered. At present, 10 months after lung surgery, he is free from recurrence.

## Discussion

In various series, clinically apparent pulmonary metastases were found in 5-27% of prostate cancer patients [2,3]. These metastases are usually seen only after bone metastases, and generally present in one of two basic radiological patterns [1,4]. A diffuse interstitial pattern representing lymphatic spread is the most common, but a multinodular pattern representing hematological spread may be seen on 8 to 20% of positive radiographs [2,4]. Solitary pulmonary nodules have been reported but are extremely rare [5,6].

This is the third reported case of solitary pulmonary metastasis from prostate cancer [5,6]. Those two reported patients underwent pulmonary resection for metastatic adenocarcinoma. In the present patient, the prostate cancer was what might be called a pleomorphic carcinoma composed of a mixture of spindle cell carcinoma and adenocarcinoma, an extremely rare histological type in prostate cancer.

Since the sarcomatoid component that metastasized to the lung was undifferentiated in this patient, it was immunohistochemically negative for PSA. Unlike in ordinary prostate cancer, the serum PSA level was not correlated with the progression status of the sarcomatoid component. These findings suggest that the sarcomatoid component did not produce PSA at all.

Hormone therapy had been effective for the adenocarcinoma component; however, the sarcomatoid component did not show sensitivity to hormone therapy. In addition, the sarcomatoid component by itself might be more invasive than normal adenocarcinoma. The tumor cells in the posterior invasion area were predominant with the more invasive sarcomatoid cancer. Thus, the sarcomatoid cancer might have survived and metastasized to the lung. The mainstay of treatment for patients with pulmonary metastases, as with other sites of metastasis, is hormonal therapy [2,7]. The pulmonary metastatic lesion in this patient tended to enlarge during hormone therapy. This unresponsiveness to medical therapy led us to consider surgical treatment.

As is evident from the absence of PSA production and unresponsiveness to hormone therapy, the sarcomatoid component of a prostate tumor may have a biological behavior different from that of prostate adenocarcinoma. Solitary nodular metastasis of prostate cancer is extremely rare, and might develop due to the biological differences of sarcomatoid cancer. Thus, we believe that surgical resection may be applicable for solitary pulmonary metastasis of this sarcomatoid cancer.

## Consent

Written informed consent was obtained from the patient for the publication of this case presentation and accompanying images. A copy of the written consent is available for review by the Editor-in-Chief of this journal.

## Abbreviations

CT: computed tomography; PSA: prostate-specific antigen

## Competing interests

The authors declare that they have no competing interests.

## Authors' contributions

TG wrote the manuscript. TG and RK performed surgery. AM carried out the pathological examination. YO and RK were involved in the final editing. All authors have approved the final manuscript.
